# Cryopreservation of infectious *Cryptosporidium parvum* oocysts achieved through vitrification using high aspect ratio specimen containers

**DOI:** 10.1038/s41598-020-68643-6

**Published:** 2020-07-16

**Authors:** Justyna J. Jaskiewicz, Derin Sevenler, Anisa A. Swei, Giovanni Widmer, Mehmet Toner, Saul Tzipori, Rebecca D. Sandlin

**Affiliations:** 1 Department of Infectious Disease and Global Health, Cummings School of Veterinary Medicine, Tufts University, North Grafton, MA USA; 2 Center for Engineering in Medicine, Department of Surgery, Massachusetts General Hospital, Harvard Medical School, and Shriners Hospitals for Children, Boston, MA USA

**Keywords:** Microbiology, Engineering

## Abstract

Infection with protozoa of the genus *Cryptosporidium* is a leading cause of child morbidity and mortality associated with diarrhea in the developing world. Research on this parasite has been impeded by many technical limitations, including the lack of cryopreservation methods. While cryopreservation of *Cryptosporidium* oocysts by vitrification was recently achieved, the method is restricted to small sample volumes, thereby limiting widespread implementation of this procedure. Here, a second-generation method is described for cryopreservation of *C. parvum* oocysts by vitrification using custom high aspect ratio specimen containers, which enable a 100-fold increase in sample volume compared to previous methods. Oocysts cryopreserved using the described protocol exhibit high viability, maintain in vitro infectivity, and are infectious to interferon-gamma (IFN-γ) knockout mice. Importantly, the course of the infection is comparable to that observed in mice infected with unfrozen oocysts. Vitrification of *C. parvum* oocysts in larger volumes will expedite progress of research by enabling the sharing of isolates among different laboratories and the standardization of clinical trials.

## Introduction

Cryptosporidiosis is a leading cause of diarrheal disease and infant death in low- and middle-income countries. The Global Enteric Multicenter Study identified infection with *Cryptosporidium* parasites as the second most common cause of moderate-to-severe diarrhea in infants in Sub-Saharan Africa and South-East Asia^1^. In this setting, cryptosporidiosis is strongly associated with death of infants and toddlers^1,2^. In fact, acute cryptosporidiosis caused > 48,000 deaths among children below the age of five in 2016 alone, according to the Global Burden of Disease Study^3^. Long-term consequences of early childhood cryptosporidiosis are related to malabsorption and undernutrition and include cognitive impairment and stunted growth^4,5^. It is estimated that burden from cryptosporidiosis, adjusted for undernutrition, accounted for more than 12 million disability-adjusted life-years in 2016^6^. Additionally, cryptosporidiosis is also a key opportunistic infection among immunocompromised individuals, responsible for life-threatening persistent diarrhea^7^. In industrialized countries, prevalence of cryptosporidiosis is on the rise with a 13% increase in the number of outbreaks per year in the USA^8^. Current anti-parasitic drugs are ineffective and no vaccine is available to control the disease. These trends highlight the need to develop vaccines, better therapeutics and diagnostic tools for cryptosporidiosis. Until recently, a major technical limitation to research on cryptosporidiosis was the lack of methods for cryopreserving the parasite. In the absence of such methods, laboratory strains of *Cryptosporidium* are continuously propagated in laboratory animals (calves, pigs and rodents) every 6–8 weeks. This process is expensive and time-consuming, and has for years limited the sharing of well-characterized isolates among laboratories. This further precludes execution of clinical trials with standardized parasite preparations and hinders the development and evaluation of therapeutics and vaccines.

Scientists have attempted to cryopreserve infectious *Cryptosporidium* oocysts for decades without much success^9–14^. These poor outcomes may be due in part to insufficient loading of cryoprotective agents (CPAs, e.g. dimethylsulfoxide (DMSO), ethylene glycol, etc.). Specifically, the complex tetralaminar oocyst wall shielding sporozoites, the fragile invasive stage of the parasite, also prevents effective CPA loading^15^. Recently, our group reported a method to permeabilize the oocyst wall using hypochlorite, which enabled uptake of CPAs without compromising oocyst viability^16^. This treatment led to the development of a protocol for cryopreservation of infectious *C. parvum* oocysts by vitrification, an ice-free method of cryopreservation^16^. While conventional slow-cooling approaches to cryopreservation were attempted with CPA-loaded oocysts, thawed parasites were not infectious to mice, which is consistent with insufficient CPA uptake. In contrast, oocysts that were cryopreserved by apparent vitrification using the ultra-rapid cooling method yielded motile sporozoites of normal morphology, and they were infectious to interferon-gamma (IFN-γ) knockout mice.

Vitrification, unlike slow-cooling, utilizes high concentrations of CPAs (~ 4–8 M) combined with rapid cooling rates to achieve formation of an amorphous glassy state that is devoid of ice crystals. Given the toxicity of CPAs, the vitrification field has moved toward the development of containers that enable extremely rapid cooling rates, where the geometry of the specimen container can be manipulated to reduce the thermal mass of the sample, thereby enabling more rapid cooling rates. As the CPA concentration necessary to vitrify is inversely related to the cooling rate, these containers enable vitrification with significantly lower CPA concentrations^17–22^. Our group, in particular, utilized this approach for the vitrification of *Cryptosporidium* oocysts, where silica microcapillaries with an internal diameter of 200 µm were used to achieve a cooling rate of ~ 250,000 °C/min^16^. However, the dimensions of the microcapillary limit the sample volume to 2 µL and permit cryostorage of only 10^5^ oocysts per device. While this cryopreservation protocol addresses a major bottleneck in the study of *Cryptosporidium*, and serves as proof of concept that oocysts are amenable to vitrification, the volume restriction and cumbersome methodology limit its practicality for routine laboratory use. Ideally, an oocyst cryopreservation method should enable storage of multiple inocula originating from a single sample in order to ensure uniformity for testing of vaccine and therapeutic candidates in pre-clinical and clinical trials.

Here, we report a cryopreservation protocol that enables increased sample volume through the design and manufacture of novel high aspect ratio specimen containers referred to as ‘vitrification cassettes’. This strategy increases the volume of a single cryopreserved sample by 100-fold up to ca. 200 µL compared to the previously reported microcapillaries. This volume is sufficient to produce multiple inocula from a single cryopreserved sample (e.g. 4 doses for pigs or > 20 human doses for clinical trials), thus overcoming a major bottleneck in the development and testing of therapeutics for the treatment of cryptosporidiosis.

## Results

### Oocysts cryopreserved in commercially available containers are not viable or infectious

To enable cryopreservation of increased sample volumes, a range of commercially available specimen containers that allow ≥ 200 µL were initially tested including cryovials and insemination straws for compatibility with the previously developed CPA loading protocol^16^, with modifications (Supplementary Fig. S1, Supplementary Table S1). Specifically, as the thermal mass of the sample is increased in these larger specimen containers, the CPA concentration must also be increased to facilitate vitrification. This was accomplished by increasing the concentration of DMSO from 30 to 40%. The CPA solution was further supplemented with polyvinylpyrrolidone (PVP), an extracellular CPA demonstrated to reduce ice crystallization during thawing. When this CPA solution of DMSO and PVP was loaded into the specimen containers and submerged in liquid nitrogen, visual examination indicated a vitreous state was achieved. Although oocyst viability assessed by propidium iodide (PI) exclusion ranged between 25–35% (35.5 ± 3.4% for cryovial and 25.3 ± 7.1% for insemination straw) after thawing, oocysts failed to infect IFN-γ knockout mice (Supplementary Figs. S1b,c). This failure is likely due to damage induced by the formation of lethal intracellular ice crystals as a consequence of insufficient CPA concentration within the oocyst. Specifically, as the cooling rate is reduced to ~ 10^3^ °C/min^23^, a higher concentration of CPA is required to prevent ice crystal formation inside of the oocysts. Our next efforts therefore focused on both increasing the intracellular CPA concentration within oocysts and increasing cooling rates while maintaining a storage volume of ≥ 200 µL.

### Development of high aspect ratio specimen containers to enable vitrification of bulk volumes

**Figure 1:**
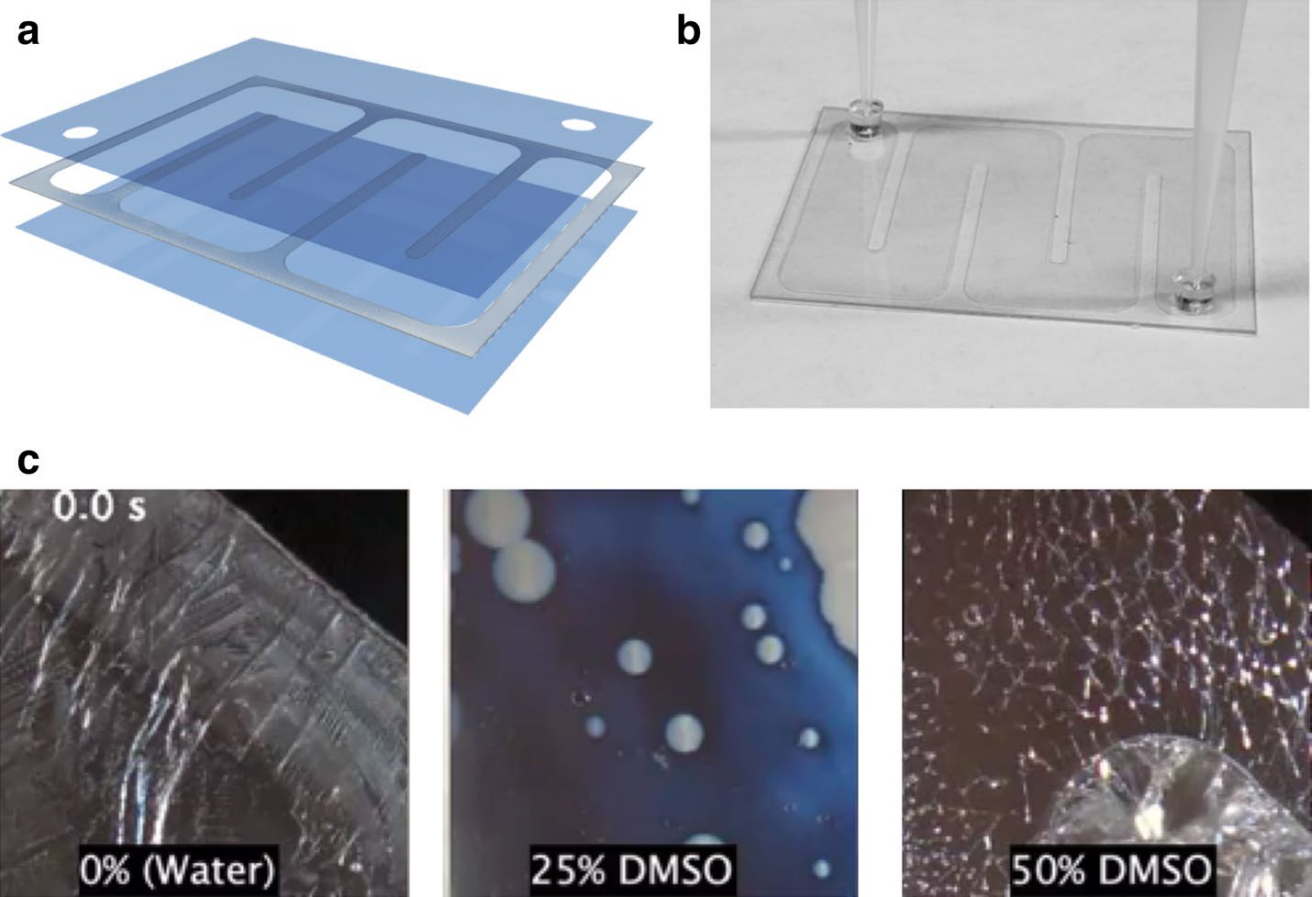
Design of high aspect ratio vitrification cassette. (**a**) The vitrification cassette consists of two sheets of polycarbonate laminates (thickness 178 µm) bonded by a sheet of pressure sensitive adhesive (thickness 120 µm) which is cut out to form the chamber. Outer dimensions are 52 mm by 40 mm, with internal volume of ~ 200 µL. (**b**) Solutions are loaded into the cassette via pipette tip affixed to a PDMS punch (4 mm outer and 1 mm inner dimeter), which seals the cassette port and creates a vacuum. (**c**) Formation of vitreous or crystallized state is determined by visual inspection. Images reflect contents of cassettes at × 20 magnification immediately upon removal from liquid nitrogen. Pure water appears opaque indicating ice formation. A solution of 25% DMSO appears to vitrify, though ice formation occurs during transfer from liquid nitrogen to the microscope stage appearing as opaque blotches in the image. 50% DMSO appears to vitrify, though cracks form during the transfer from liquid nitrogen to the microscope stage. Supplementary Video S1 shows time-lapse of melting.

The need to maintain high cooling rates while simultaneously increasing sample volume led us to consider high aspect ratio specimen containers. We initially adopted a commercially available borosilicate glass rectangular capillary that holds a volume of up to 40 µL, but it was very fragile and shattered easily (Supplementary Fig. S2a). Next, a polycarbonate vitrification cassette based on a high aspect ratio model was designed (Fig. 1a,b, Supplementary Fig. S2b and S3). This cassette increases the sample volume to approximately 200 µL with minimal reduction of the cooling rate, estimated at 10^4^ °C/min when plunged into liquid nitrogen, based on extrapolation from the observation of partial vitrification of a 25% v/v DMSO/water solution^24,25^. Given that a minimum of 30% DMSO is necessary to form extracellular glass in vitrification cassettes, we selected 50% DMSO to maximize glass formation within the oocyst since the intracellular concentration of CPA is likely much lower than that present in the extracellular solution (Fig. 1c and Supplementary Video S1). Strategies to increase the concentration of intracellular CPA, such that conditions for vitrification are created inside of the oocysts, were next optimized.

### Optimization of hypochlorite treatment improves CPA uptake

**Figure 2:**
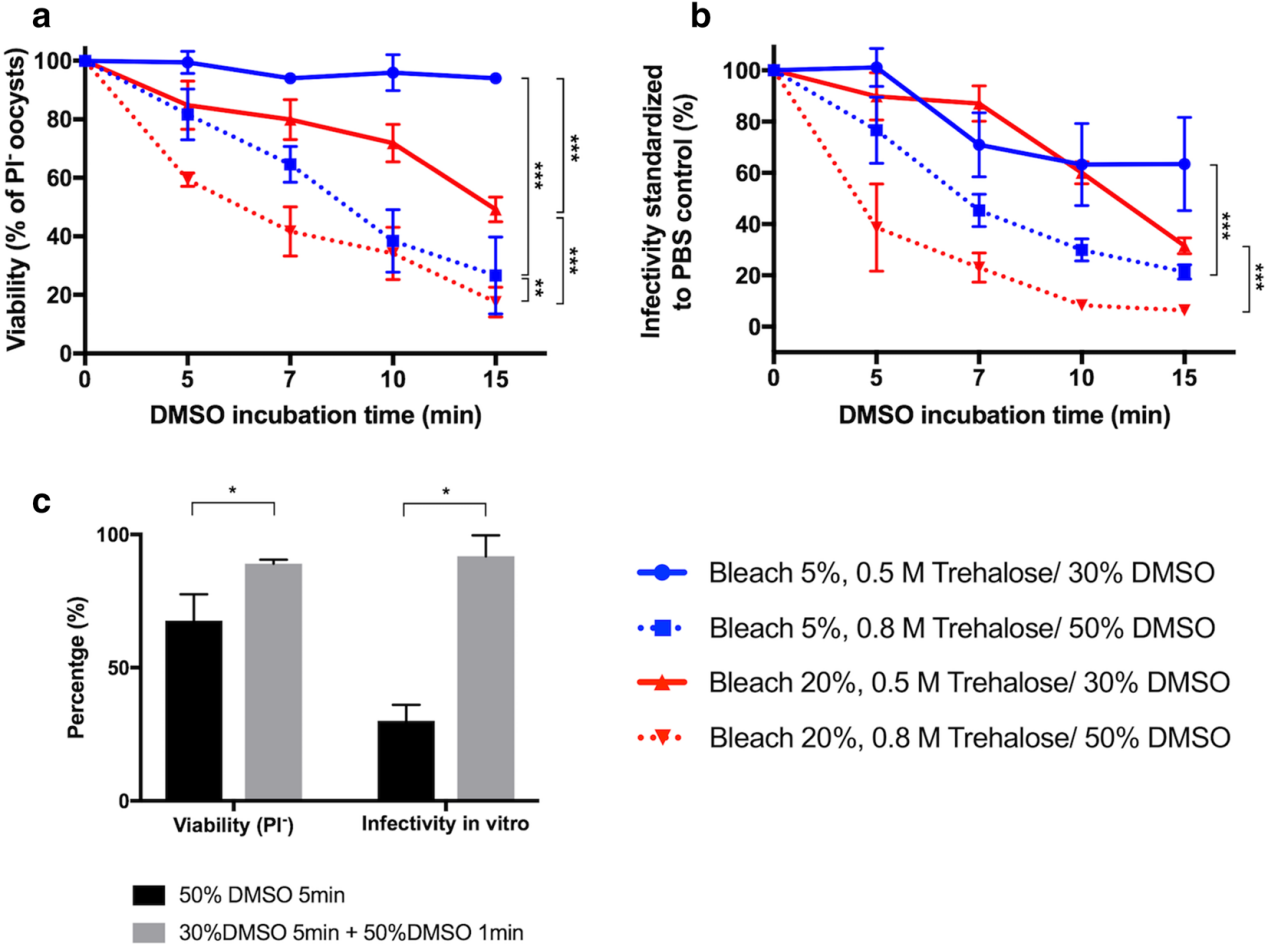
Approaches to increase intracellular concentration of CPA. Increasing concentrations of bleach, trehalose and DMSO were applied to maximize intracellular permeation of CPA among *C. parvum* oocysts. (**a**) Viability of oocysts permeabilized with either 5% or 20% bleach, followed by incubation with a CPA cocktail containing varying concentrations of trehalose and DMSO over a duration of 5–15 min. Viability was measured by PI exclusion after removal of CPA (n = 3). Differences in viability in response to bleaching and trehalose/DMSO treatment were measured using two-way ANOVA (***p < 0.0001; **p = 0.002). (**b**) Infectivity was measured as percent of intracellular parasitic stages established in MDBK cells in comparison to control oocysts incubated with PBS in lieu of DMSO (n = 4). Differences in infectivity in response to DMSO concentration were measured for both permeabilization/ dehydration protocols using two-way ANOVA (***p < 0.0001). (**c**) Reduced CPA toxicity was accomplished through the introduction of a two-step CPA loading protocol. Toxicity due to CPA alone (pre-freeze) was measured in terms of viability and in vitro infectivity following treatment with 50% DMSO in either one- or two-step additions. Oocysts were bleached (20%), dehydrated (1.6 M trehalose, 10 min) and then incubated with 50% DMSO for 5 min or for 1 min following 5 min 30% DMSO incubation. Viability is reported as percent of PI^−^ oocysts and infectivity as percent of intracellular parasitic stages established in MDBK cells in comparison to control oocysts incubated with PBS in lieu of DMSO (n = 3). Differences between treatments were measured using Mann–Whitney test (*p = 0.05). All values indicate means and error bars indicate standard deviation.

Given the impermeable nature of the oocyst wall, an approach to increase CPA uptake is through improved permeabilization. Previously, treatment with hypochlorite was found to enable partial uptake of CPAs^16^. Trehalose was then added to induce osmotic dehydration of oocysts, thereby increasing the overall concentration of CPAs within the oocyst and reducing the risk of formation of intracellular ice crystals. Here, we attempted to increase this effect by applying harsher bleaching conditions and increasing the osmotic trehalose gradient. Oocysts permeabilized by bleach and dehydrated in trehalose, both in increasing concentrations, were subsequently exposed to varying concentrations of DMSO (30% and 50%) over a 15 min period and evaluated for viability as an indirect measure of CPA uptake. Figure 2a,b illustrate the fitness of oocysts based on their viability and infectivity in vitro following CPA removal and recovery in PBS. Harsher bleaching conditions were found to increase permeability of oocysts to DMSO, both at 30% and 50% concentration, as evidenced indirectly by an observed increase in mortality rates. Oocysts incubated with 50% DMSO maintained acceptable viability and in vitro infectivity during 5 min of incubation (for 20% bleach condition: 59.4 ± 2.3% viability, 38.7 ± 17.0% in vitro infectivity). However, after 15 min or longer, DMSO at this concentration was found to be highly toxic (for 20% bleach condition: 17.5 ± 5.0% viability and 6.4 ± 1.9% infectivity). Oocysts exposed to the cocktail of 0.8 M trehalose/50% DMSO for 5 min and then subsequently cooled using both types of high aspect ratio specimen containers (i.e. rectangular capillary or cassette), were not infectious to IFN-γ knockout mice (Supplementary Fig. S2c, Supplementary Table S1). This indicates that 50% DMSO, although necessary for vitrification in these specimen containers, accumulates significant pre-freeze and post-thaw cytotoxicity, rendering oocysts non-infectious.

### Optimization of the CPA loading procedure for compatibility with vitrification cassettes

To ensure glass formation in the vitrification cassettes, we next focused on increasing the intracellular concentration of DMSO while minimizing cytotoxicity. This was accomplished by adding DMSO in a step-wise manner rather than all at once, such that a final extracellular concentration of 50% is achieved with reduced toxicity. In this approach, oocysts were first incubated in 30% DMSO for 5 min, and then 50% DMSO for one additional minute. This significantly increased pre-freeze viability and infectivity in vitro in comparison to the one-step DMSO addition at the same overall concentration (Fig. 2c). Based on low oocyst permeability, we expect that for the two-step protocol, the additional DMSO (50% v/v) is unlikely to accumulate substantially inside the oocyst as the duration of exposure is limited to 1 min. However, based on previous observations^16^, oocysts are expected to substantially dehydrate in response to the hyperosmotic extracellular gradient created by extracellular DMSO. Thus, the initial low concentration of DMSO within the oocyst is expected to increase as a result of water loss caused by the osmotic gradient applied during the second CPA loading step. Given the minimal toxicity, the two-step DMSO loading protocol was prioritized for the cryopreservation of oocysts using the vitrification cassettes.

### Cryopreservation in vitrification cassettes yields oocysts that exhibit in vitro infectivity

**Figure 3:**
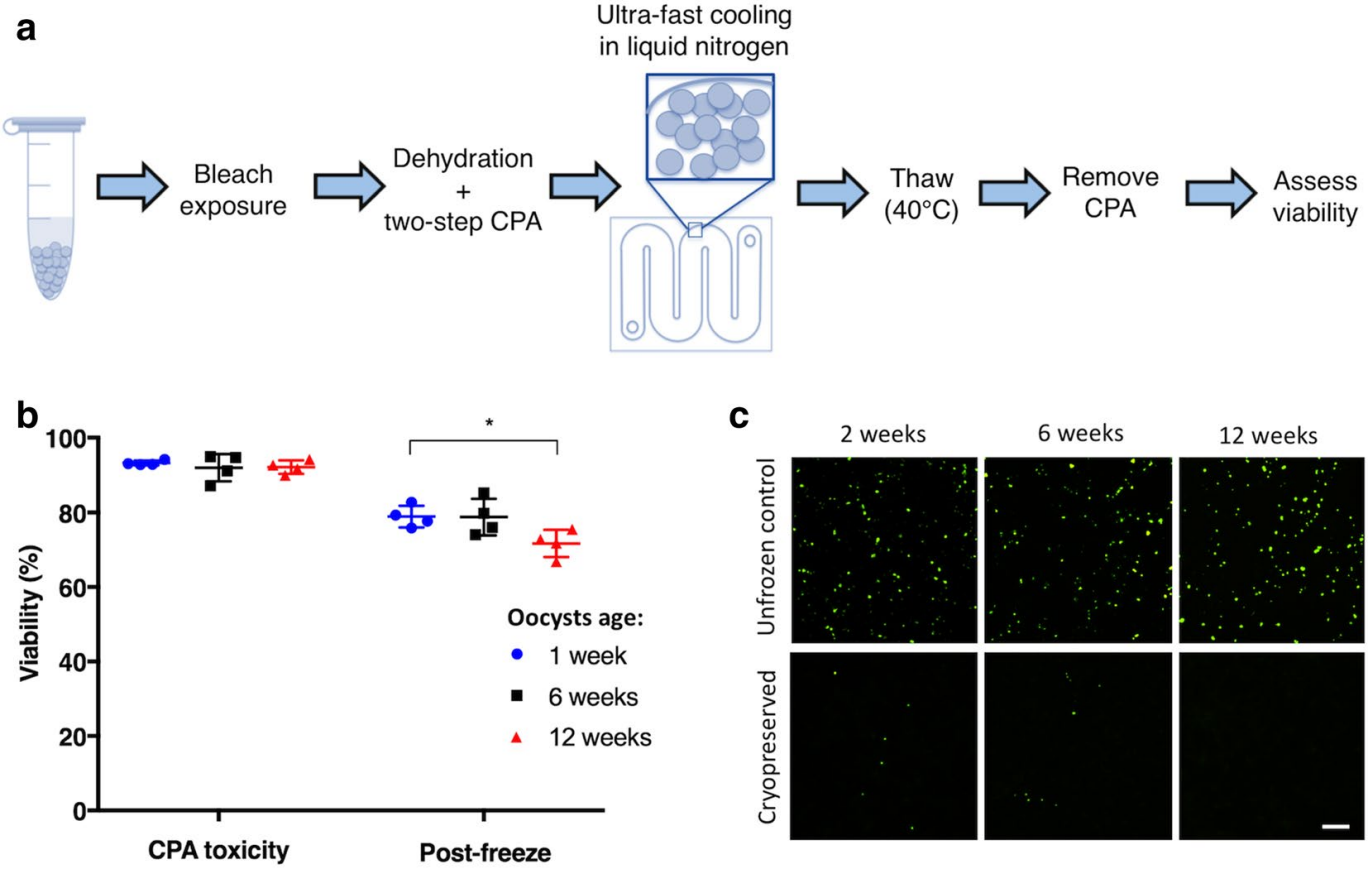
Oocysts frozen in vitrification cassettes are viable and exhibit in vitro infectivity. (**a**) *C. parvum* oocysts at 1, 6 and 12 weeks of age were bleached (20%), dehydrated in 1.6 M trehalose and then incubated with 30% DMSO for 5 min and additional 50% DMSO for 1 min. Oocysts were then immediately loaded into cassettes and submerged in liquid nitrogen for 10 min to achieve vitrification. Oocysts were thawed by quickly transferring the cassette from liquid nitrogen to a 40 °C water bath. Viability and infectivity were quantified after CPA removal. (**b**) Oocyst viability was determined by PI exclusion, both pre-freeze and after thawing of oocysts at different ages. Horizontal lines indicate mean and error bars standard deviation (n = 4). There were no differences in pre-freeze and thawed (post-freeze) viability between oocysts of different ages (Kruskal–Wallis test, pre-freeze: *p* = 0.64; post-freeze: *p* = 0.06), except a slight decrease in viability after thawing for 12- in comparison to 1-week-old oocysts (Mann–Whitney test, *p = 0.03). (**c**) In vitro oocyst infectivity was determined using the MDBK infection assay. Thawed oocysts were co-incubated with MDBK cells for 24 h at MOI 1:1. Establishment of intracellular stages was detected using FITC-labeled *Vicia villosa* lectin and imaged under × 200 magnification. Scale indicates 50 µm. Additional images are available in Supplementary Fig. S4.

Since previous work revealed the importance of oocyst age on cryopreservation outcome^16^, the present protocol was tested on a single batch of oocysts at different ages: 1, 6 and 12 weeks. Figure 3a shows the workflow used in these vitrification experiments (for detailed protocol, see Supplementary Protocol S1). First, oocysts were permeabilized by treatment with 20% bleach for 1 min and then suspended in 1.6 M trehalose for 10 min to promote dehydration. Next, a final concentration of 50% DMSO/0.5 M trehalose was achieved using the stepwise CPA loading protocol described above. The sample was then loaded into a vitrification cassette and rapidly submerged in liquid nitrogen for 10 min to ensure the specimen was fully vitrified. While 10 min under liquid nitrogen may be brief, this is sufficient time for the specimen to equilibrate at − 196 °C. At this temperature, enzymatic activity essentially stops, and therefore the quality of the specimen at 10 min is reflective of the quality expected under much longer storage durations. The sample was later thawed by brief submersion in a 40 °C water bath. Figure 3b shows the toxicity associated with CPA exposure for oocysts of different ages, as compared to the post-thaw toxicity caused cumulatively by CPA exposure and cryopreservation. Cryopreservation yielded 70–80% viable oocysts based on PI exclusion. While CPA toxicity alone reduced viability to ~ 92% regardless of oocyst age, the oldest oocysts exhibited significantly increased mortality after thawing (78.9 ± 2.5%, 78.8 ± 4.2% and 71.7 ± 3.1% at 1-, 6- and 12-weeks, respectively). As exclusion of PI alone is found to be an inaccurate marker of viability^26,27^, oocyst viability testing was coupled with the in vitro infectivity assay. Cryopreserved oocysts were found to invade and establish intracellular stages inside MDBK cells, with the exception of 12-week-old oocysts (Fig. 3c, Supplementary Fig. S4), which failed to invade. This compares favorably to the previously observed trend, where oocysts cryopreserved at an older age exhibit reduced infectivity in animals^16^.

### Cryopreservation in vitrification cassettes yields oocysts that are infectious in vivo

**Figure 4:**
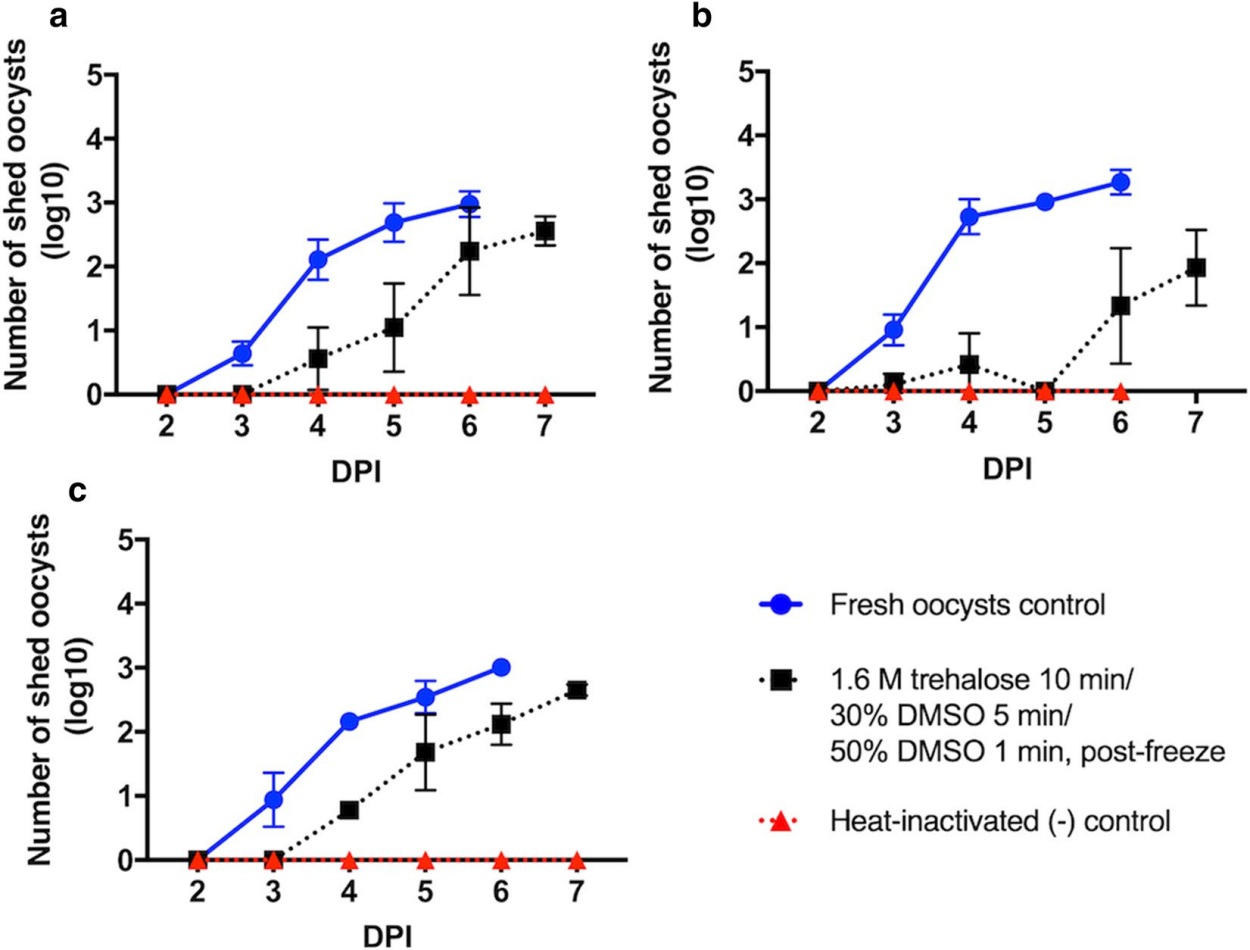
Cryopreserved *C. parvum* oocysts are infectious to IFN-γ knockout mice. Bleached (20%) and dehydrated (1.6 M trehalose, 10 min) *C. parvum* oocysts were frozen at the age of 2, 6 and 12 weeks, using vitrification cassettes after application of the two-step DMSO loading protocol, such that oocysts were first incubated in 30% DMSO for 5 min, followed by 1 min incubation in 50% DMSO. Oocysts were submerged in liquid nitrogen for 10 min, which is sufficient to ensure complete equilibration under cryogenic conditions. IFN-γ knockout mice (n = 3) were then inoculated orally with 30,000 PI^−^ thawed or unfrozen control oocysts. Intensity of fecal shedding was quantified daily by microscopic enumeration of oocysts in 30 fields of acid-fast stained fecal smears under × 1,000 magnification. Positive (unfrozen) and negative (heat-inactivated) oocyst controls were included as age-matched controls. To determine whether oocyst age affects survival during cryopreservation, 2- (**a**), 6- (**b**) and 12-week-old (**c**) oocysts were studied. Values indicate means of log transformed oocysts count and error bars indicate standard deviation, except for 4 dpi for 12-week old oocysts, which indicates a pooled sample. Viability of cryopreserved oocysts was determined by PI exclusion prior to inoculation and was as follows: 75.9%, 74.1% and 76% for 2-, 6- and 12-week-old oocysts, respectively. Untransformed data can be found in Supplementary Fig. S5a–c.

Based on the promising results obtained from in vitro testing, the infectivity of thawed oocysts was assessed using the IFN-γ knockout mouse model. Figure 4a–c show that oocysts of different ages (2, 6 and 12 weeks) cryopreserved in liquid nitrogen for 10 min using the vitrification cassettes were infectious to mice. Specifically, mice inoculated with cryopreserved oocysts developed a patent infection at 4 days post infection (DPI), a day later than mice inoculated with unfrozen control oocysts, as evidenced by onset of fecal oocyst shedding. Contrary to our in vitro findings, no reduction in oocyst infectivity in vivo as a function of oocyst age was observed, as inferred from the onset of oocyst shedding on the same day. This data demonstrates that oocysts aged up to 12 weeks can be successfully cryopreserved using vitrification cassettes. We estimate that ~ 10^7^ oocysts can be obtained from one mouse infected with cryopreserved oocysts by direct purification from intestines collected *post mortem*. Additionally, collection of feces throughout the course of infection can further maximize the total output of oocysts.

## Discussion

Here, cryopreservation of *C. parvum* oocysts was achieved by incubation of permeabilized oocysts in an optimized CPA cocktail containing DMSO and trehalose combined with application of ultra-fast cooling rates, facilitated by vitrification cassettes. The geometry of the cassette was designed to maintain a rapid cooling rate despite the increased sample volume of 200 µL. Such increased volume comes inherently with a reduction in cooling rate in comparison to that of the previously reported microcapillary used for vitrification of oocysts^16^. We balanced the conditions of glass formation at this lower cooling rate by increasing the concentration of trehalose and DMSO in the extracellular solution and the intracellular compartment under conditions of increased oocyst permeability. Interestingly, *Cryptosporidium* parasites possess a synthetic pathway for trehalose^28^. We suspect that the native presence of this known glass-forming disaccharide^29,30^ may further benefit the cryopreservation outcome by promotion of intracellular glass formation upon dehydration of the oocyst.

Notably, a 200 µL storage volume allowed by the vitrification cassette enables storage of ca. ~ 6,000,000 oocysts in a single specimen container. For comparison, cryopreservation of the same number of oocysts using our previously described method would require over 60 individually loaded microcapillaries, therefore entailing significantly more time and labor. Overall, the vitrification cassettes enable cryopreservation of oocysts with reduced labor and technical challenges, which is likely to facilitate broader dissemination of the protocol. This is particularly useful as the field invests resources into genome sequencing^31,32^ and genetic modification^33,34^, where cryopreservation of well characterized strains becomes an urgent priority.

The ability to cryopreserve large batches of oocysts may be transformative to the field by enabling broader access to parasites for research. Drug testing efforts and vaccine development would also benefit considerably from the availability of cryopreserved *Cryptosporidium* reference lines. Ideally, inocula for drug or vaccine trials should be of the same optimized batch to ensure reproducibility. Cryopreservation in one container eliminates variability expected with the use of multiple microcapillaries as each container undergoes separate cooling and rewarming events. Importantly, one vitrification cassette can hold a sufficient number of oocysts to produce multiple inocula from a single cryopreserved sample (e.g. ~ 4 doses for piglets or > 20 doses for human subjects), whereas ~ 20 microcapillaries would be required to produce a single inoculum for a piglet. Additionally, studies involving human subjects should be of the same standardized and validated batch of oocysts. Our cryopreservation protocol can reduce the effort of standardization and validation of specimens in vitro and in vivo following each propagation prior to evaluation at different testing sites. The ability to cryopreserve large numbers of oocysts from a single batch and thaw as needed can ascertain uniformity of the challenge dose and thus reduce considerably batch-to-batch variation. We recognize a key limitation of this study is the minimal number of strains validated. While the previously developed method of ultra-rapid vitrification has now been successfully applied to different isolates of *C. parvum*, *C. hominis* and *C. tyzzeri*, including transgenic strains, the method examined here must be evaluated further to ensure robustness of the protocol.

Vitrification with reduced CPA concentrations and ultra-rapid cooling rates has been shown to be effective for the preservation of many cell types. However, this approach characteristically comes at the cost of sample volume. Here, we have described a novel high aspect ratio vitrification cassette that strikes the balance between the competing requirements for maximal surface area to volume ratio and sufficient specimen volumes for future clinical and research applications.

## Material and methods

### Oocyst sources and collection method

Fresh *C. parvum* oocysts (MD isolate of cervine origin passaged repeatedly in sheep and mice^35^) were generated at the Tufts University by infection of four 8-week-old female CD-1 mice (Charles River Laboratories, Wilmington, MA). Propagation of oocysts in mice was conducted in an institution accredited by the Association for Assessment and Accreditation of Laboratory Animal Care (AAALAC) in compliance with the study protocol No. G2017-107 approved by the Tufts University Institutional Animal Care and Use Committee (IACUC) in accordance with the Guide for the Care and Use of Laboratory Animals (National Research Council).

Mice were grouped in bedded microisolators with access to food and water ad libitum. Mice were immunosuppressed throughout the course of experiment by administration of dexamethasone in drinking water at a concentration 16 mg/L. After three days of immunosuppressive treatment, each mouse received 50,000 oocysts in a PBS suspension by oral gavage. Starting from 5 DPI, which marks onset of fecal oocyst shedding, mice were kept in collection microisolators during the night for collection of fecal material and then rested in bedded microisolators during the day, both with access to food and water ad libitum. At termination of the experiment on 15 DPI, mice were euthanized by carbon dioxide asphyxiation, followed by cervical dislocation as required by the Tufts University IACUC guidelines. Oocysts were purified from mice feces by ether extraction and separated on a nycodenz gradient, as described elsewhere^36^ with minor modifications. Briefly, homogenized fecal slurry was filtered through a 100 µm cell strainer and treated with diethyl ether in water containing 0.5% Tween 80 at the ratio 2:3. Oocysts were isolated by ultracentrifugation (13,400×*g*, 60 min) on a 10%/25% nycodenz gradient. Harvested oocysts were then resuspended in PBS and stored in 4 °C. For consecutive propagation of fresh oocysts, we utilized intestines collected post-mortem from the positive control IFN-γ knockout mice (Jackson Laboratory, Bar Harbor, ME, USA) infected with unfrozen oocysts. Briefly, at the completion of the experiment, a section of gut was excised between duodenal bulb and rectum. Guts were homogenized with Tissue Master 125 homogenizer (Omni International, Kennesaw, GA, USA). Homogenized gut slurry was then processed as described above for fecal slurry with the exception of the filtration step using a cell strainer.

### Oocyst bleaching

Oocysts were bleached by incubation with either 5% or 20% dilutions of commercial bleach containing 8.25% sodium hypochlorite (Clorox Original, The Clorox Company, CA, USA). Following incubation with the indicated concentration of bleach on ice for 1 min, oocysts were then washed three times in PBS followed by centrifugation (18,000×*g*, 2 min) after each wash to remove supernatant.

### Cell lines

Madin-Darby Bovine Kidney (MDBK) cells (ATCC CCL-22) were maintained in the CO_2_ incubator at 37 °C in RPMI media (Gibco) containing 10% heat-inactivated (56 °C, 30 min) fetal bovine serum (FBS), 2 mM l-glutamine (Cellgro) and 100 U/penicillin/50 µg/mL streptomycin sulfate solution (MP Biomedicals). Cells were passaged weekly by serial subculture. Briefly, the cell monolayer was detached by incubation with 0.05% trypsin and 0.02% EDTA solution (Invitrogen) at 37 °C in the CO_2_ incubator for 7 min. Collected cells were washed with fresh media, of which 10% was subcultured into the new flask.

### CPA pre-freeze toxicity in vitro

#### Viability testing

In order to increase the intracellular concentration of CPA, oocysts were bleached with various concentrations of bleach—5% or 20% and then incubated with CPA cocktails consisting of trehalose (Sigma) and DMSO (Sigma) at different concentrations. Briefly, bleached oocysts were first dehydrated in 1.6 M or 1 M trehalose solution for 10 min and then incubated for another 5–15 min with DMSO achieving its final concentration of 30% or 50% (ambient conditions were used throughout). The DMSO/trehalose cocktail was then diluted by incubation with excess PBS (at ratio 1:100) for 30 min at room temperature and removed by centrifugation (18,000×*g*, 2 min). Cytotoxicity attributable to the CPA cocktail was measured by inclusion of PI (Sigma) at 10 µg/mL using flow cytometry (Accuri C6, BD Life Sciences) and was normalized to its control in PBS for each condition.

In an effort to maximize the concentration of intracellular and extracellular CPA without compromising oocyst viability, a protocol was optimized in which oocysts were gradually exposed to higher concentrations of DMSO by two-step addition. Here, 6-week-old bleached oocysts (5% or 20%) were first dehydrated in 1.6 M trehalose for 10 min. DMSO was added to achieve 0.8 M trehalose/30% DMSO solution and incubated for 5 min. The second addition of DMSO led to a final concentration of 0.5 M trehalose/50% DMSO and was incubated for 1 min. CPA cocktail was then diluted as described above. Cytotoxicity attributable to CPA cocktail was measured by inclusion of PI and was normalized to the untreated oocyst control in PBS.

#### Infectivity testing in vitro

All CPA cocktails described in Fig. 2a were further tested for infectivity in the in vitro model of epithelial cell infection using MDBK cells. Twenty thousand cells were seeded into each well of a 96-well culturing plate (Falcon) and maintained for two days to reach confluence (defined at 4 × 10^4^/well). Oocysts were prepared for infection by bleaching and incubation with CPA cocktail as described above. After removal of diluted CPA in excess PBS, the oocyst pellet was resuspended in 50 µL of 0.75% sodium taurocholate (Sigma) and incubated at 37 °C for 15 min to prompt excystation. Pre-excysted oocysts were then added onto confluent cell monolayers at MOI 1:3. After 24-h co-incubation of parasite with cell monolayer, cultures were washed with PBS to remove extracellular stages of parasite, permeabilized with 100% methanol for 10 min at room temperature and then washed again with PBS. After fixation, cells were blocked with 10% FBS for 30 min and then incubated with fluorescein-labeled *Vicia villosa* lectin (Vector Laboratories) at concentration 1 µg/mL for 30 min to visualize intracellular stages of the parasite. After the final wash with PBS, monolayers were inspected with a Nikon Eclipse Ti-E microscope (Nikon Instruments Inc.). Four images were taken of each well at 200 × magnification using a FITC filter at 100 ms exposure. The number of fluorescing infection foci was then counted by ImageJ 1.48v particle analyzer^37^ using the following settings: triangle threshold 15–255, size exclusion at 10–1,000 pixel^2^ and circularity at 0.1–1.0. Results depicted in Fig. 2b are expressed as percent infectivity of the control oocysts, which were treated with PBS in place of CPA.

### Vitrification cassettes

The vitrification cassette consists of two sheets of polycarbonate laminates (thickness 178 µm) bonded by a sheet of pressure sensitive adhesive (thickness 120 µm) which is cut out to form a chamber. The top sheet of polycarbonate has two holes on either end of the chamber for sample loading and unloading. Sample is loaded using two 0.2–1 mL pipette tips fixed with punched PDMS gaskets with 4 mm outer and 1 mm inner diameter (Drummond Scientific). The chamber is shaped in a serpentine to avoid trapping bubbles during sample loading, and to improve sample recovery by flushing. The chamber outer dimensions are 52 mm by 40 mm and internal volume approximately 200 µL (Supplementary Fig. S3). The cassettes can be produced in large volumes by existing reel-to-reel manufacturing processes. Fabrication services were provided by Grace BioLabs (Bend, OR, USA) using an AutoCAD file as a template (Supplementary File S1).

Rapid cooling in the cassettes was validated by imaging at 20 × magnification. Water-DMSO mixtures were rapidly plunged in liquid nitrogen, then removed and imaged under a stereomicroscope Zeiss Stemi 508 (Carl Zeiss Microscopy, Germany) while gradually rewarming. Ice crystallization during gradual warming in air (transition from transparent to opaque white) is observed > 25% v/v DMSO, indicating successful vitrification.

### Cryopreservation protocols for ultra-rapid cooling of oocysts

#### Cryopreservation using larger commercially available specimen containers

The vitrification protocol previously reported using microcapillaries^16^ was applied with modifications to larger containers such as polyethylene terephthalate insemination straw (Cryo Bio System, France) and an ultra-clear USP class IV polycarbonate cryovial (VWR). The concentration of DMSO was increased from 30 to 40% to account for decreased rate of temperature transfer as a result of an increase in container diameter and wall thickness. Additionally, polyvinylpyrrolidone (PVP) (Sigma) was added to DMSO in order to ensure glass formation and prevent ice crystallization upon thawing. Briefly, bleached oocysts (5%, 1 min) were incubated in 1 M trehalose for 10 min. A cocktail of DMSO and PVP was then added to achieve a final concentration of 0.5 M trehalose/6% PVP/40% DMSO and incubated for 5 min at room temperature. Fifty µL aliquots were then frozen in an insemination straw or a cryovial by rapidly plunging in liquid nitrogen and then thawed in a 40 °C water bath for 30 s. Expelled contents were incubated in excess PBS and processed as described above.

#### Cryopreservation using novel high aspect ratio specimen containers

Briefly, 2,000,000 *C. parvum* MD oocysts were treated with 20% Clorox bleach for 1 min on ice and then washed with PBS. Oocysts were centrifuged (18,000×*g*, 2 min), resuspended in 20 µL of 1.6 M trehalose and incubated at room temperature for 10 min. Next, 20 µL of 60% DMSO was added to dehydrated oocysts to achieve the concentration of 0.8 M trehalose/30% DMSO and was incubated for 5 min at room temperature. Lastly, 20 µL of 90% DMSO was added to achieve final concentration of 0.5 M trehalose/50% DMSO. Following the second addition of DMSO, oocysts were loaded into polycarbonate cassettes (Grace Biolabs #RD500011; 52 mm × 40 mm) in the following manner: 60 µL of oocysts in CPA cocktail was loaded onto the cassette port and was aspirated into the cassette via negative pressure created by removing the air from the opposite port with 1 mL pipette tip fixed with punched PDMS gasket (Drummond Scientific). While the present study only utilized 60 µL inocula containing 2,000,000 oocysts at a time, a single cassette can hold more than three times this volume. Loaded cassettes were then rapidly submerged in liquid nitrogen such that exposure to 50% DMSO did not exceed 1 min. Although the sample reaches cryogenic temperatures nearly instantaneously, oocysts were maintained in liquid nitrogen for 10 min prior to thawing. Thawing was achieved by quickly transferring the cassette from liquid nitrogen to a 40 °C water bath for 20 s. It is critical that the cassette be rapidly transferred into liquid nitrogen, as well as be rapidly transferred from liquid nitrogen to 40 °C for thawing, in order to avoid lethal ice crystallization. Contents of the cassette were expelled using hydraulic pressure applied by flushing PBS through the cassette port sealed with PDMS gasket attached to the 1 mL pipette tip. The contents expelled through the opposite port were collected with pipette and transferred to 1.5 mL tube to incubate in excess PBS for 30 min at room temperature. Supernatant was then removed from samples by centrifugation (18,000×*g*, 2 min) and 100 µL of PBS was used to resuspend the pellet. Thawed oocysts were stored on ice prior to further testing.

Modification of the protocol described above with a one-step DMSO addition, was also tested using rectangular capillaries and vitrification cassettes. Here, oocysts were incubated in 1.6 M trehalose for 10 min and then in DMSO for 5 min achieving final concentration of 0.8 M trehalose/50% DMSO, before being frozen in liquid nitrogen for 10 min using either rectangular borosilicate glass capillaries (VitroTubes 0.2 × 2 mm, Vitrocom) or polycarbonate cassettes, as described above. Samples were then thawed in 40 °C water bath for 20 s. Expelled contents were incubated in excess PBS and processed as described above.

### In vitro viability and infectivity of cryopreserved oocysts

Bleached (20%) *C. parvum* oocysts at 1–2, 6 and 12 weeks of age were cryopreserved as described above using a two-step DMSO addition protocol, such that final concentration of 0.5 M trehalose/50% DMSO was achieved. Oocysts exposed to cryoprotective cocktails but not frozen were also studied to discern lethality due to CPA toxicity rather than freezing. Pre-freeze and post-freeze oocyst viability was measured by PI exclusion at 10 µg/mL using flow cytometry and by an in vitro infectivity assay. Briefly, MDBK cells were prepared for infection as described above. Cells were infected with cryopreserved oocysts at MOI 1:1. After 24 h incubation with parasites, cells were processed following the immunofluorescence protocol as described above in order to detect intracellular stages of the parasite. Micrographs of infection were taken under 200 × magnification using FITC filter at 100 ms exposure (Nikon Instruments Inc.).

For evaluation of cryopreservation in insemination straws and cryovials, pre-freeze and post-freeze viability was measured only by PI exclusion as described above.

### In vivo infectivity of cryopreserved oocysts

*C. parvum* oocysts at 2, 6 and 12 weeks of age were bleached (20%) and cryopreserved in the vitrification cassettes as described above using a two-step DMSO addition protocol with final concentration of 0.5 M trehalose/50% DMSO. Infectivity of cryopreserved oocysts was evaluated in 8-week-old female IFN-γ knockout mice bred under pathogen-free conditions (Jackson Laboratory, Bar Harbor, ME, USA). All mice experiments were conducted in an AAALAC accredited institution in compliance with the study protocol No. G2017-107 approved by the Tufts University Institutional Animal Care and Use Committee in accordance with the Guide for the Care and Use of Laboratory Animals (National Research Council). Mice were randomly assigned into groups of three by a blinded animal caretaker, however the investigators were not blinded during this experiment.

The animal care, inoculation and monitoring procedures were performed as described elsewhere^16^, with modifications. Mice were immunosuppressed throughout the course of the experiment by administration of dexamethasone in drinking water at concentration 10 mg/L. Three days after immunosuppressive treatment started, each mouse received 30,000 PI^−^ cryopreserved oocysts in PBS suspension by oral gavage. Each batch of experiments was performed in the presence of a positive control group (mice inoculated with 30,000 fresh PI^−^ oocysts) and a negative control group (mice inoculated with 30,000 heat-inactivated oocysts). For evaluation of fecal oocyst shedding, feces were collected daily from each mouse individually starting from 3 DPI. Mice demonstrating evidence of fecal oocyst shedding were euthanized by carbon dioxide asphyxiation followed by cervical translocation on 7 DPI, unless prematurely terminated due to recumbency. Mice negative for signs of infection were monitored until 11 DPI. Intensity of fecal shedding was measured daily by inspection of the acid-fast stained fecal smear slides, prepared from a single fecal pellet of approximately equal size, for presence of oocysts in 30 fields (1000 ×).

The animal experiments which produced negative cryopreservation outcome were performed as described above with exception of few procedural details. A summary table describing the different cryopreservation methods evaluated and the corresponding outcome can be found in Supplementary Table S1. Briefly, for evaluation of one-step DMSO cryopreservation protocol in vitrification cassettes and rectangular capillaries, 6- and 2-week-old oocysts were used, respectively, and animals were infected with a dose of 10,000 PI^−^ oocysts. For evaluation of cryopreservation outcome in insemination straws and cryovials, 8-week-old oocysts were used and mice received a dose of 5,000 PI^−^ oocysts. Though we inoculated mice with a higher number of oocysts using the protocol described for vitrification cassettes, we believe positive infection was due to improvement of the cryopreservation protocol compared to other methods that yielded negative outcome, due to the observation that the course of infection between thawed and untreated oocysts were similar.

### Statistical analysis

All in vitro infection experiments were performed in two technical repeats and were replicated three or four times to ensure reproducibility. All in vitro viability experiments were performed in triplicate. For in vivo experiments groups of 3 animals were used to assess oocyst infectivity. Graphing and statistical analyses of data were performed using GraphPad Prism software (v7.0c, GraphPad Software, Inc.).

## Supplementary information

Supplementary File S1.

Supplementary Information 1.

Supplementary Video S1.

## Data Availability

The datasets generated during this study are available from the corresponding author on reasonable request.
