# Supplementary material for: Cryopreservation of infectious Cryptosporidium parvum oocysts achieved through vitrification using high aspect ratio specimen containers
Source: Sci Rep. 2020 Jul 16;10:11711. doi: 10.1038/s41598-020-68643-6 (PMC7366687; doi:10.1038/s41598-020-68643-6)
Supplement: Supplementary file 2 — Supplementary Information 1. [file 41598_2020_68643_MOESM2_ESM.docx]

Supplementary information

Cryopreservation of infectious *Cryptosporidium parvum* oocysts achieved through vitrification using high aspect ratio specimen containers

Justyna J. Jaskiewicz^1^, Derin Sevenler^2^, Anisa A. Swei^2^, Giovanni Widmer^1^, Mehmet Toner^2^, Saul Tzipori^1*^, Rebecca D. Sandlin^2*^

^1^ Department of Infectious Disease and Global Health, Cummings School of Veterinary Medicine, Tufts University, North Grafton, MA, USA.

^2^ Center for Engineering in Medicine, Department of Surgery, Massachusetts General Hospital, Harvard Medical School, and Shriners Hospitals for Children, Boston, USA.

^*^Corresponding author:

E-mail: [saultzipori@tufts.edu](mailto:saultzipori@tufts.edu) (ST) or [rdsandlin@mgh.harvard.edu](mailto:rdsandlin@mgh.harvard.edu) (RS)

**
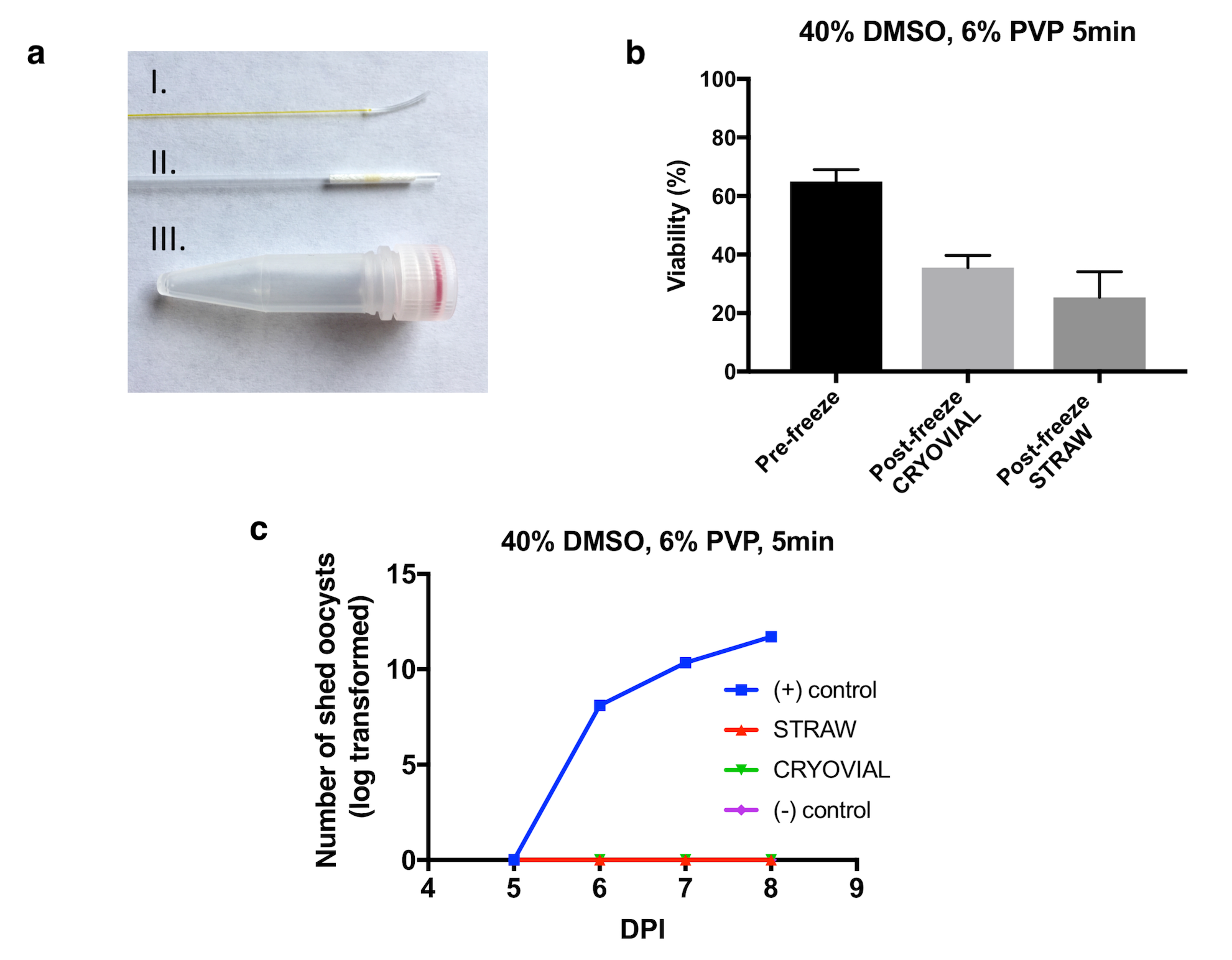
**

**Supplementary Figure S1. Cryopreservation in commercial devices does not preserve viable and infectious oocysts.**  The protocol previously optimized for cryopreservation of oocysts using microcapillaries [1] was applied with modifications to larger specimen containers. Although viable oocysts were observed after thawing, they were not infectious to animals. **(a)** Specimen containers are shown for comparison to the microcapillary (I) including an insemination straw (II) and cryovial (III). **(b)** Viability was measured by propidium iodide (PI) exclusion after incubation of oocysts with a cocktail of 40% DMSO/ 6% PVP for 5 min, without freezing (CPA toxicity control) or after thawing in liquid nitrogen using a cryovial or an insemination straw (n=3). **(c)** Oocysts cryopreserved in liquid nitrogen using insemination straws or cryovials after 5 min incubation with 40% DMSO / 6% PVP are not infectious to interferon-gamma (IFN-γ) knockout mice (n=3, mice were inoculated orally with 5,000 PI- oocysts). Positive (unfrozen) and negative (heat-inactivated) oocyst treatments were included as matched controls. Intensity of fecal shedding was quantified daily by microscopic enumeration of oocysts in 30 fields of acid-fast stained fecal smears under 1000x magnification. Values indicate means of log transformed oocysts count and error bars indicate standard deviation. Viability of oocysts was determined by PI exclusion prior to inoculation and was as follows: 93.9%, 33.8%, 40.3% and 5.3% for fresh control, oocysts frozen in a straw, oocysts frozen in a cryovial and the (-) control, respectively.

**
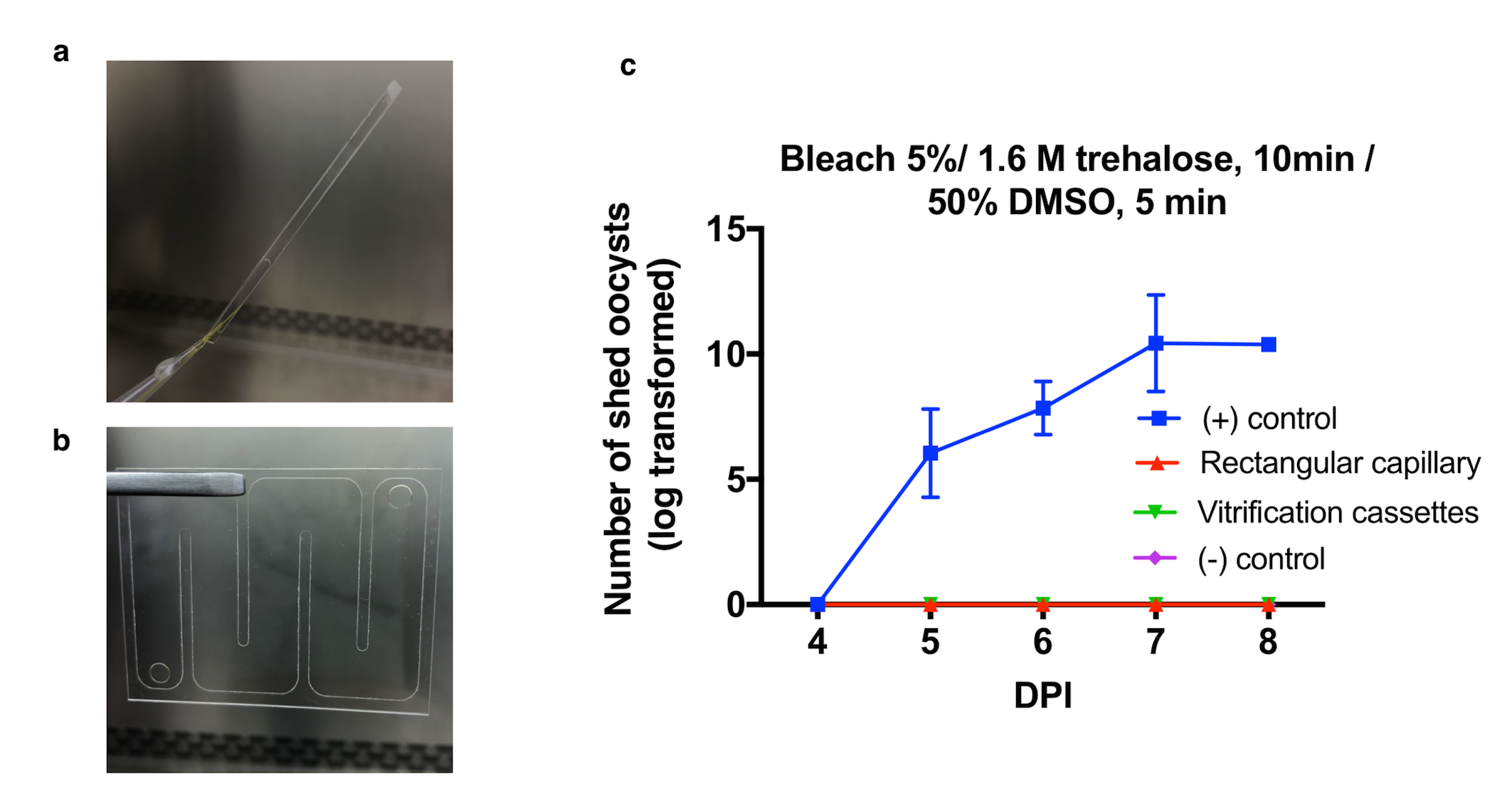
**

**Supplementary Figure S2. Cryopreservation in high aspect ratio specimen containers after one-step DMSO addition does not preserve infectious oocysts.** Oocysts cryopreserved in high aspect ratio specimen containers: (a) rectangular capillary and (b) vitrification cassette after one-step addition of 50% DMSO (5 min incubation) did not produce infection in IFN-γ knockout mice. **(c)** Mice were inoculated orally with 10,000 PI- oocysts. Unfrozen and heat-inactivated oocysts were included as matched positive and negative control, respectively. Intensity of fecal shedding was quantified daily by microscopic enumeration of oocysts in 30 fields of acid-fast stained fecal smears under 1000x magnification. Values indicate means of log transformed oocysts count and error bars indicate standard deviation. Viability of oocysts was determined by PI exclusion prior to inoculation and was as follows: 98%, 59.9%, 63.0% and 2.4% for fresh control, oocysts frozen in a rectangular capillary, oocysts frozen in cassettes and the (-) control, respectively.


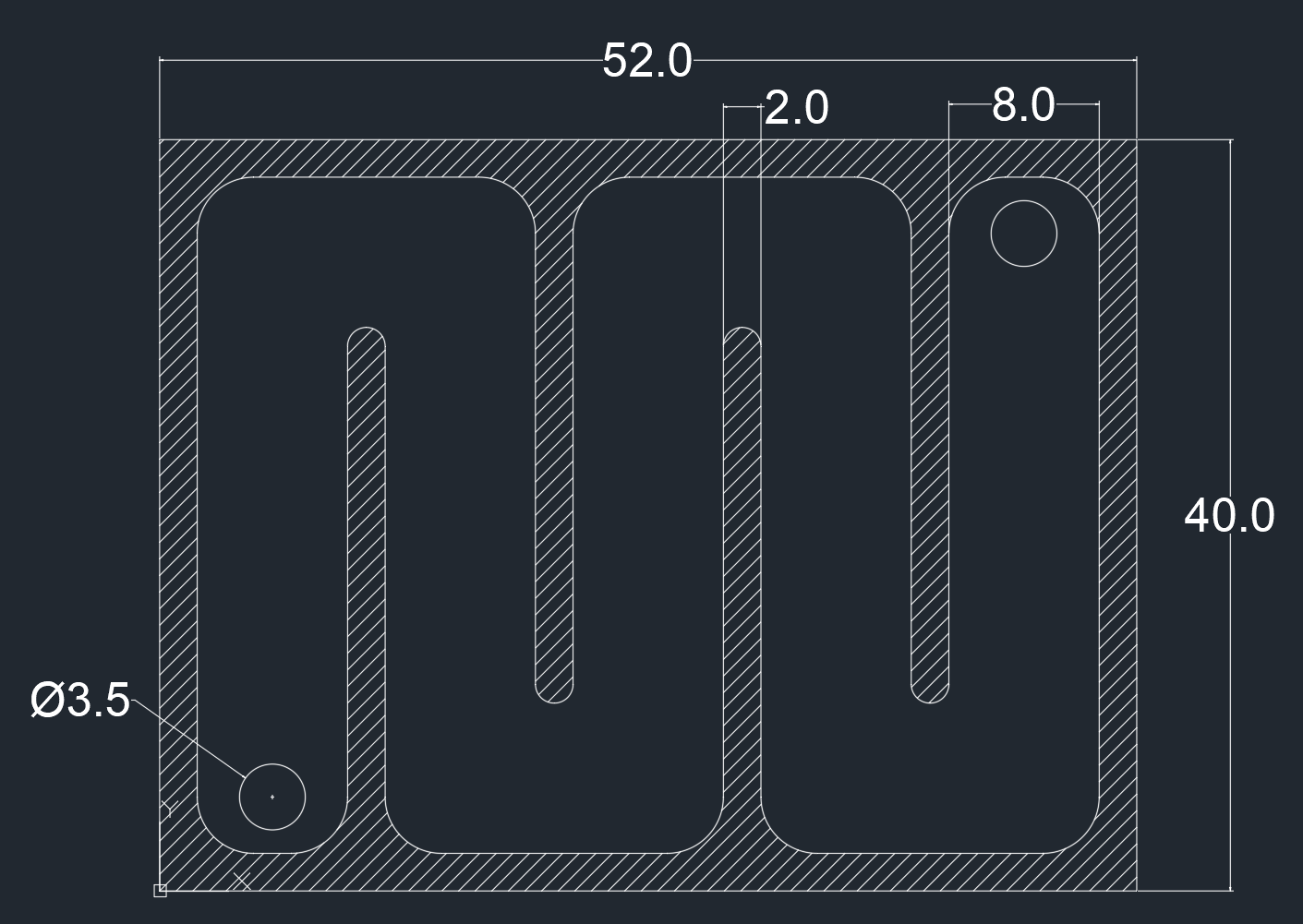


**Supplementary Figure S3. Dimensions of the vitrification cassette in millimeters.**


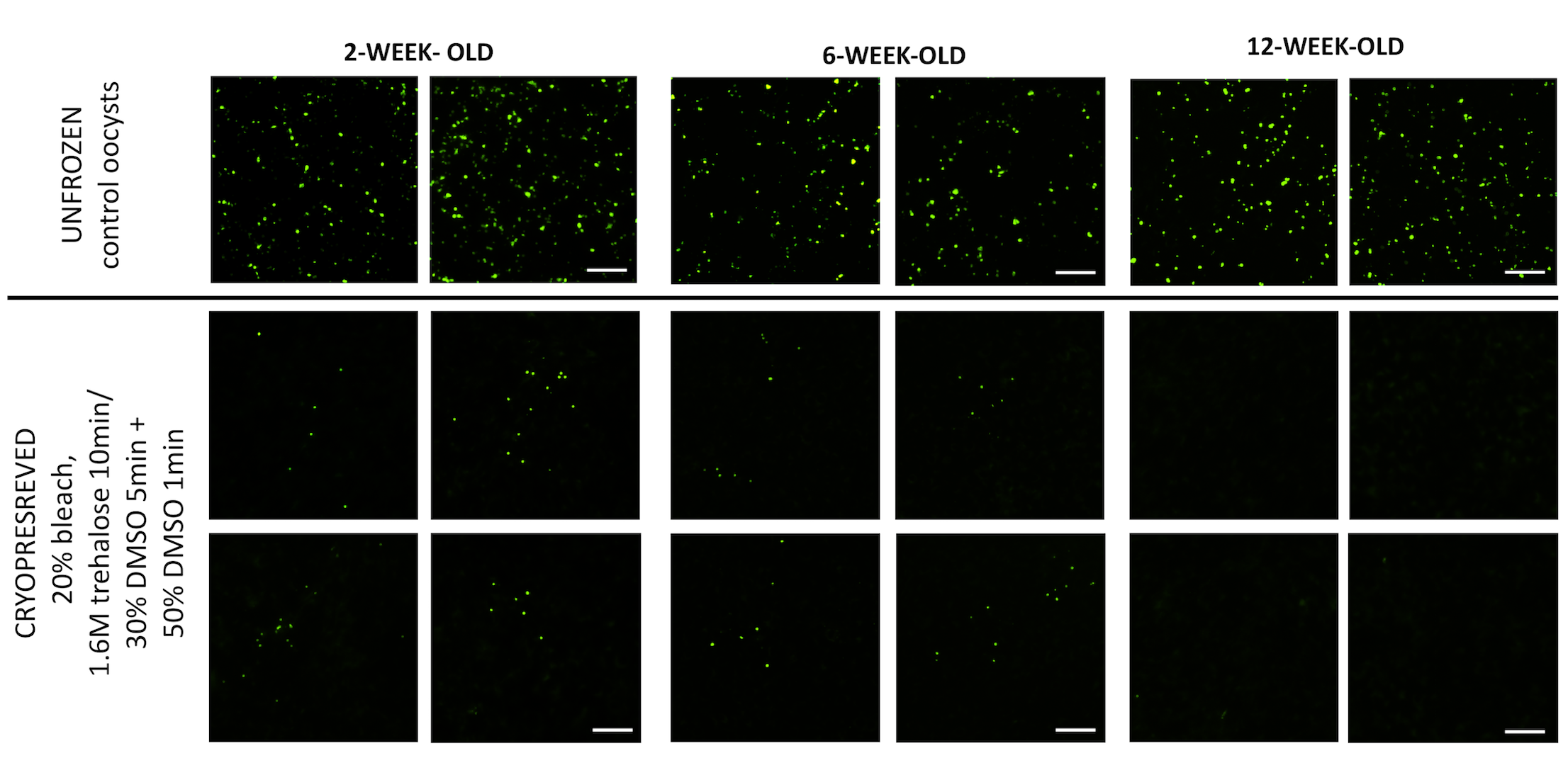


**Supplementary Figure S4. Cryopreservation in vitrification cassettes yields infectious oocysts *in vitro*.** Oocysts cryopreserved in vitrification cassettes using a two-step DMSO addition protocol are infectious to MDBK cells *in vitro*, with exception of oocysts preserved at the age of 12 weeks. Thawed oocysts were co-incubated with MDBK cells for 24h at MOI 1:1. Establishment of intracellular stages was detected using FITC-labeled *Vicia villosa* lectin and imaged under 200x magnification. Viability of thawed oocysts was determined by PI exclusion prior to inoculation and was as follows: 79.9%, 77.9% and 69.3% for 2-, 6- and 12-week-old oocysts respectively (n=3). The first panel on the left (unfrozen control) and the first panels on the top left (thawed oocysts) are depicted in Fig 3C in the main manuscript. Scale indicates 50µm.

**
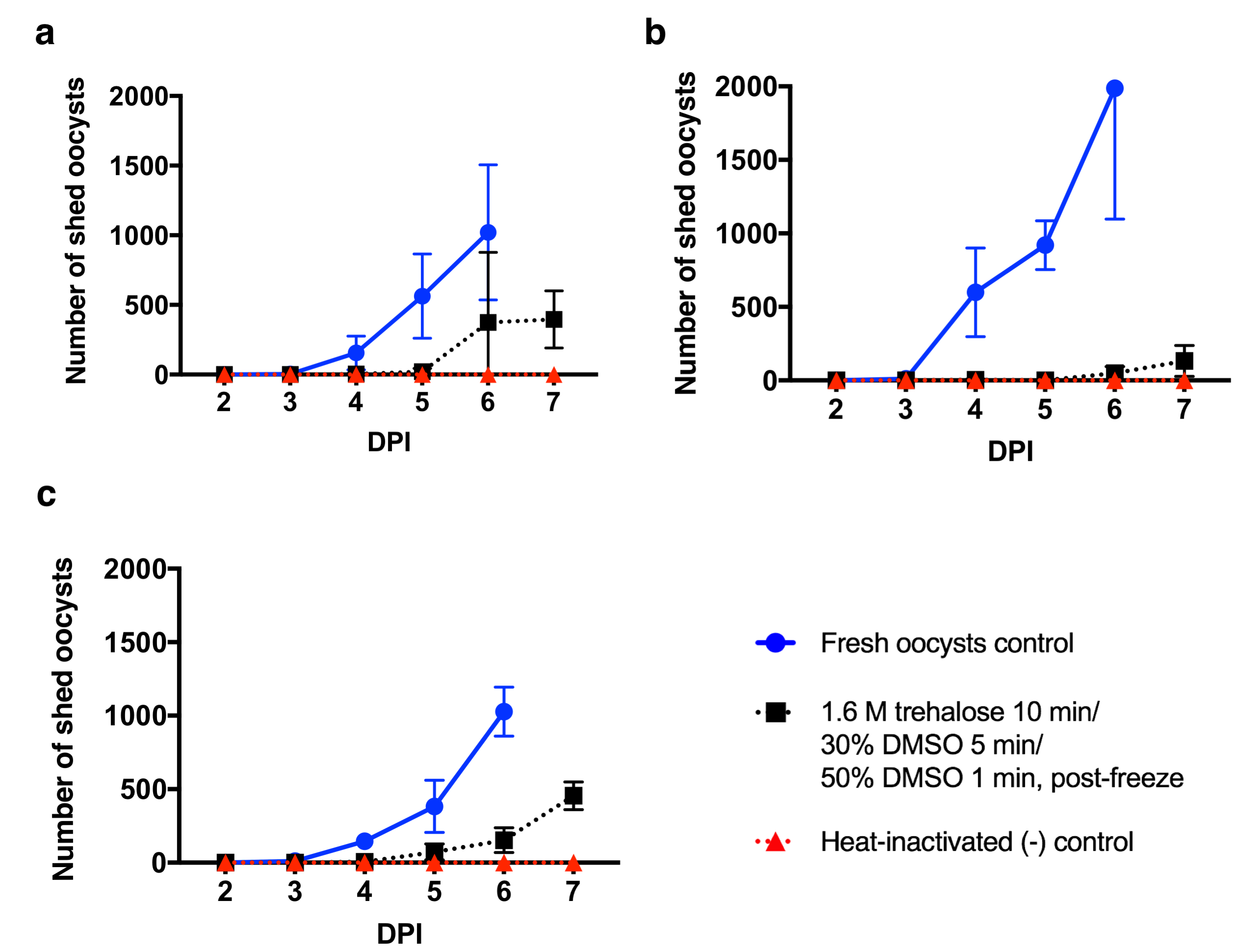
**

**Supplementary Figure S5. *C. parvum* oocysts cryopreserved in vitrification cassettes are infectious to IFN-γ knockout mice (untransformed data).** Bleached (20%) and dehydrated (1.6 M trehalose, 10 min) *C. parvum* oocysts were cryopreserved at the age of 2, 6 and 12 weeks, using vitrification cassettes after application of the two-step DMSO addition protocol, such that oocysts were first incubated in 30% DMSO for 5 min, followed by 1 min incubation in 50% DMSO. Oocysts were then rapidly submerged in liquid nitrogen for 10 min. IFN-γ knockout mice (n=3) were inoculated orally with 30,000 PI^-^ thawed or unfrozen control oocysts. Intensity of fecal shedding was quantified daily by microscopic enumeration of oocysts in 30 fields of acid-fast stained fecal smears under 1000x magnification. Unfrozen and heat-inactivated oocysts were included as age-matched positive and negative control, respectively. To determine whether oocyst age affects survival using the cryopreservation protocol, 2- **(a)**, 6- **(b)** and 12-week-old **(c)** oocysts originating from a single batch were studied. Values indicate means of log transformed oocyst counts and error bars indicate standard deviation, except for 4 dpi for 12-week old oocysts, which indicates a pooled sample. Viability of cryopreserved oocysts was determined by PI exclusion prior to inoculation and was as follows: 75.9%, 74.1% and 76% for 2-, 6- and 12-week-old oocysts, respectively.

**Supplementary Table S1. Summary of cryopreservation protocols and their outcome in animal models.**

| Specimen  Container | Age of  oocysts | Concentration  of trehalose  (initial/final) | DMSO  addition  protocol | CPA  concentration | DMSO  incubation  time | Inoculation  dose | Infectivity in  animals after  thawing |
| --- | --- | --- | --- | --- | --- | --- | --- |
| Insemination  straw | 8 weeks | 1 M / 0.5 M | One-step | 40% DMSO  (+ 6% PVP) | 5 min | 5,000 | No |
| Cryovial | 8 weeks | 1 M / 0.5 M | One-step | 40% DMSO  (+ 6% PVP) | 5 min | 5,000 | No |
| Rectangular capillary | 2 weeks | 1.6 M / 0.8 M | One-step | 50% DMSO | 5 min | 10,000 | No |
| Vitrification cassette | 6 weeks | 1.6 M / 0.8 M | One-step | 50% DMSO | 5 min | 10,000 | No |
| Vitrification cassette | 2-12  weeks | 1.6 M / 0.5 M | Two-step | 30% DMSO/  50% DMSO | 5 min/  1 min | 30,000 | Yes |

**Supplementary Protocol S1. Ultra-fast cooling of oocysts using vitrification cassettes.**

The method of ultra-fast cooling in vitrification cassettes reported here was demonstrated to be a robust method to cryopreserve oocysts using two-step DMSO addition protocol. The method is however extremely sensitive to variations in technique. Below, we provide a more detailed protocol including potential missteps. Splashing may occur while using liquid nitrogen, thus special attention to safety is critical, including but not limited to the use of face shield and cryoprotective gloves. While the protocol below is standardized for freezing oocysts in 60 µL volume, the cassette can accommodate 200 µL.

1. Bleaching: 2 million oocysts (in 100 µL PBS contained in a 1.5 mL Eppendorf tube) of 2-12-week-old *C. parvum* (MD isolate) were treated with 100µL of 40% Clorox bleach in PBS (20% final concentration) and incubated on ice for 1 min. The solution was then centrifuged at 18,000xg for 1 min (Note: For volumes >500 µL the centrifugation time is increased to 2 min). Supernatant was immediately removed and 1 mL of PBS added, followed by an additional 2 min centrifugation. This washing step is repeated three times to entirely remove bleach from the solution. Following the final washing step, the supernatant was completely removed in preparation for step #2.

2. The packed oocyst pellet was suspended in 20 µL of 1.6 M trehalose solution (prepared in PBS) and incubated at ambient temperature for 10 min.

3. 20 µL of a 60% v/v solution of DMSO (prepared in PBS) was then added to the oocysts to achieve a final concentration of 0.8 M trehalose/30% DMSO and incubated at ambient temperature for 5 min.

4. 20µL of a 90% v/v solution of DMSO (prepared in PBS) was then added to the oocysts to achieve a final concentration of 0.5 M trehalose/50% DMSO.

5. Oocysts were then immediately loaded into a vitrification cassette in the following manner: 60 µL of oocysts in CPA cocktail was loaded onto the cassette port and was aspirated into the cassette via negative pressure created by removing the air from the cassette via the opposite port using 1 mL pipette tip affixed to a punched PDMS gasket, such that the gasket seals the port, as demonstrated in Fig 1b. Loading was performed within 1 min in order to minimize DMSO permeation, as longer durations of exposure are expected to damage sporozoites. The cassette was placed on a clean Petri dish during loading.

6. Freezing: Using tweezers, the cassette was placed perpendicular to the liquid nitrogen (contained within a Styrofoam box). Using a steady but very rapid movement, the cassette was then plunged into the liquid nitrogen. This ensures even heat transfer through the sample and is likely critical to achieve the positive cryopreservation outcome. Based on our experience with other applications, fluctuations in technique during the freezing and thawing steps are the most common reasons for poor cryopreservation outcome.

7. Thawing: using tweezers, the cassette was transferred in a steady but rapid movement into a 40ºC water bath and allowed to thaw for 20 sec. Care should be taken here as increased exposure to the 40ºC water bath is expected to induce spontaneous excystation of sporozoites from oocysts.

8. To harvest oocysts, the cassette was placed on a clean Petri dish, port-side up. Contents of the cassette were expelled using hydraulic pressure applied by flushing PBS through the cassette port using 1 mL pipette tip affixed to a PDMS gasket which seals the port. The content expelled through the opposite port was collected with pipette and transferred to 1.5 mL tube. Approximately 1mL of PBS was used to expel contents and rinse contents of one cassette. Collected oocysts solution was filled to 1.5 mL with PBS.

9. The oocyst solution was incubated in ambient temperature for 30 min to allow DMSO to exit the oocyst.

10. The oocyst solution was centrifuged at 18,000xg for 2 min.

11. The supernatant was then removed and the oocysts were resuspended in 100µL PBS, followed by evaluation of viability, excystation and infectivity.

**Supplementary Video S1. DMSO vitrifies in high aspect ratio cassettes at 50%** **v/v.** Videos show time-lapse of melting of the cassette contents at 20x magnification immediately upon removal from liquid nitrogen. Pure water appears opaque indicating ice formation. A solution of 25-40% DMSO appears to vitrify, though ice formation occurs during transfer from liquid nitrogen to the microscope stage, and appear as opaque blotches in the image. 50% DMSO appears to vitrify, though cracks form during the transfer from liquid nitrogen to the microscope stage**.**

**Supplementary File S1. AutoCAD file of the vitrification cassette.**

**References:**

1. Jaskiewicz, J. J. *et al.* Cryopreservation of infectious *Cryptosporidium parvum* oocysts. *Nature communications* **9**, 2883, doi:10.1038/s41467-018-05240-2 (2018).
